# Automatic Forest-Fire Measuring Using Ground Stations and Unmanned Aerial Systems

**DOI:** 10.3390/s110606328

**Published:** 2011-06-16

**Authors:** José Ramiro Martínez-de Dios, Luis Merino, Fernando Caballero, Anibal Ollero

**Affiliations:** 1 Robotics, Computer Vision and Control Group, Universidad de Sevilla, Seville, 41092, Spain; E-Mails: fcaballero@us.es (F.C.); aollero@cartuja.us.es (A.O.); 2 Robotics, Computer Vision and Control Group, Pablo de Olavide University, Seville, 41013, Spain; E-Mail: lmercab@upo.es; 3 Centre for Advanced Aerospace Technology (CATEC), Seville, 41309, Spain

**Keywords:** perception systems, Unmanned Aerial Systems, forest fires

## Abstract

This paper presents a novel system for automatic forest-fire measurement using cameras distributed at ground stations and mounted on Unmanned Aerial Systems (UAS). It can obtain geometrical measurements of forest fires in real-time such as the location and shape of the fire front, flame height and rate of spread, among others. Measurement of forest fires is a challenging problem that is affected by numerous potential sources of error. The proposed system addresses them by exploiting the complementarities between infrared and visual cameras located at different ground locations together with others onboard Unmanned Aerial Systems (UAS). The system applies image processing and geo-location techniques to obtain forest-fire measurements individually from each camera and then integrates the results from all the cameras using statistical data fusion techniques. The proposed system has been extensively tested and validated in close-to-operational conditions in field fire experiments with controlled safety conditions carried out in Portugal and Spain from 2001 to 2006.

## 1. Introduction

Wildfires destroy thousands of hectares each year and incur very high social, environmental and economic costs. Forest-fire fighting is a very dangerous activity that requires extensive resources and causes many casualties every year. In many cases, lack of precise information on the state and evolution of the fire front is one of the main causes of accidents.

Forest-fire fighting is traditionally based on estimations made by fire fighting experts from visual observations directly on the terrain or by analyzing data provided by sensors. These estimations are subject to a high degree of error due to human inaccuracy in the visual estimation and smoke hampering the observation of the fire. More recently, airborne systems have been used in order to provide a broader view of the fire, but the monitoring activities are still carried out based on visual estimations by experts.

Automatic forest-fire perception is a complex problem. Besides the general drawbacks that are present in perception in natural scenarios, such as uncontrollable and sudden changes in environmental conditions, there are others related to the particular characteristics of fire, including the harsh environment and the difficulties in predicting the spread of fire and smoke. Most research and development efforts devoted to automatic forest-fire perception have been focused on fire detection. Although there are a large variety of automatic detection systems, the number of automatic forest-fire measuring systems that have been tested and validated in field fires is very low.

This paper describes a forest-fire measurement system that automatically computes the evolution of the most important geometrical measurements of fire propagation in real-time, such as the position of the fire front, the rate of spread and the maximum height of the flames. The proposed system addresses the sources of error present in forest-fire measurement by exploiting complementarities between different types of cameras that are distributed on the terrain, so that they can obtain a complete and robust perception of the forest fire. The main sensors used are visual and infrared cameras located at distributed ground stations and mounted on UAS. As a secondary objective, 3D views of the fire are generated from the computed measurements. These views, compatible with ARCINFO Geographical Information System (GIS), are transmitted using a TCP/IP protocol allowing them to be visualized on the Internet. Fire brigades can use them in fire fighting planning to predict the potential evolution of the fire and determine the optimal location of fire fighting resources.

The proposed measurement system combines cameras at fixed locations, which can already be installed as part of the forest infrastructure, together with cameras onboard UAS, which provide high flexibility suitable for highly changing environments. To the best of our knowledge, the system presented in this paper is one of the first forest-fire measurement systems that integrate results from visual and infrared cameras distributed at both fixed locations and cameras onboard UAS. It has been extensively validated in forest-fire field experiments in close-to-operational conditions in which plots of land of up to 2.5 hectares were burned.

This paper is structured as follows. Section 2 describes related work in forest-fire detection and monitoring and presents the motivation and main advantages and constraints of the proposed work. Section 3 briefly describes the proposed system including the general architecture, the sensors involved and the system deployment. Section 4 focuses on the processing techniques used to extract fire measurements from each camera and Section 5 describes the multi-camera data fusion method. Section 6 shows some results obtained in field experiment. Conclusions are set out in the last section.

## 2. Related Work and Motivation

A number of systems for automatic forest-fire *detection* have been developed based on different sensors. Some of the first automatic forest-fire detection systems were based on infrared cameras capable of detecting the radiation emitted by a fire [1,2]. These systems are often on ground stations at locations with high visibility. The stations are equipped with pan and tilt units that sweep the area under surveillance at specific intervals in order to detect fires at their early stages. These detection systems require a direct view of the radiation source. In addition, they often have a high false alarm rate which constrains their use in operational conditions. Some False Alarm Reduction (FAR) systems for forest-fire detection have been developed. The FAR system described in [3] combines different sources of information including infrared, visual, meteorological and terrain contextual information.

Fire detection based on visual cameras aims to detect the smoke plume produced by the fire. They can detect fires without direct vision, e.g., behind a hill. Different approaches to smoke detection with visual images have been developed based on contrast [4], texture [5], or motion analysis through wavelet analysis [6], among others. Visual-based methods require daylight to operate and the accuracy in locating the fire is lower than in the case of infrared detection. In addition, detection systems based on Light Detection and Ranging (LIDAR) devices that identify the concentration of fire smoke particles have been developed [7–9].

Forest-fire detection based on processing satellite images has been intensively researched, see [10–13] for instance. These systems have been successfully tested in large, uniform and unpopulated regions. However, in populated areas these methods have relevant limitations. Satellite spatial and temporal resolutions involve significant detection delays that can be unsuitable in crowded areas. In addition, human activity can cause frequent false alarms that hamper the application of the systems in operational conditions.

A number of automatic detection systems based on aerial platforms have been developed. The Airborne Wildfire Intelligence System (AWIS) includes wildfire detection and mapping of the fire-front and burned area [14]. The manoeuvrability of aerial vehicles such as helicopters, with hovering and vertical takeoff and landing capabilities, makes them an ideal tool to complement fixed cameras in fire monitoring and measurement. The use of UAS in forest-fire scenarios has been demonstrated in projects such as the FiRE project [15]. In the FiRE project an ALTUS UAS, an adaptation of the Predator UAS, was demonstrated in fire detection and localisation. While the FiRE project considers one single and complex vehicle endowed with high accuracy sensors, in this paper the fire measurement system uses a team of small and simple UAS cooperating in fire perception.

In [16], the feasibility of the application of a team of small (low altitude, short endurance) UAS to cooperatively monitor and track the propagation of large forest fires is explored. The paper provides simulations using a six-degree of freedom dynamic model for the UAS and a numerical propagation model for the forest fire. However, results in actual fire fighting activities have still not been carried out. In addition, in [17] a method for the ortho-rectification of images gathered from an UAS, and their application in fire monitoring activities is presented. However, no actual fire monitoring results are described.

Recently, Wireless Sensor Networks (WSNs) have also been developed for forest-fire detection activities. In [18], a system consisting of sensors (thermo and radiation with GPS) carried by animals living in the environment is used for forest-fire detection, although no experiments are presented. In [19], the design and development of a WSN involving sensors and IP cameras is described. In [20], the problem of fire detection is modelled as a k-coverage problem in WSNs. Some algorithms are proposed to solve the problem, but results are only shown in simulations. In [21], a WSN with nodes equipped with temperature and humidity sensors were used to detect a fire and extract measurements of fire spread. Although promising, this technology still has unresolved issues such as the costs of maintaining thousands of WSN nodes deployed in forest areas and their potential pollution effects.

In [22], a fire detection method based on measuring the increase in air temperature using sonar units has been proposed. Sound wave speed is affected by air temperature. Acoustic sources distributed in the forest generate sound waves with a specific frequency. Radars at fire watchtowers continuously scan for acoustic waves. The differences in the speed of acoustic waves are used to detect fires.

Despite the large variety of forest-fire detection systems, the number of automatic forest-fire monitoring and *measuring* systems is still scarce.

Some work on measuring laboratory fires in controlled conditions have been proposed using different sensors. One basic method consists of the use of a set of thermocouples to detect the location of the fire front, see [23] for instance. The work in [24] describes a method based on linear transformations of infrared images to compute the positions and the rate of spread of a linear fire front propagating on a flat surface. The processing of multispectral infrared images to determine the fire perimeter, the ‘active’ fire line and the fire propagation direction is proposed in [25]. The system described in [26] combines infrared and visual cameras to obtain fire measurements in laboratory experiments. This work was developed and validated in laboratory fires in controlled conditions. The extension to forest fires is not addressed in these papers.

The method described in [27] uses stereo-vision units with visual cameras to obtain fire geometry measurements. It is useful in laboratory fires but does not address practical problems of real forest fires, such as smoke occluding the visual images, preventing the extraction of valid measurements.

WSNs have also been proposed for this fire monitoring. In [28], *FireWxNet* is described, consisting of a WSN designed to report weather conditions (humidity, temperature, wind speed) as well as images in fire environments. The paper shows real deployment of the WSN, which is evaluated in terms of battery performance, packet yield and information gathered.

Satellite-based fire monitoring systems, such as [29–31], have been shown to be useful for monitoring of wildland fires. Although significant advances have been carried out, the temporal and spatial resolutions are still low for accurate monitoring in cases, such as urban-wildland interface areas, where very frequent and precise measurements of fire evolution are required [32,33].

We present here a system that allows us to obtain closer views and detailed information of relevance for fire fighting activities. It can provide fire measurements with a spatial resolution lower than one meter at rates of 2 Hz. Thus, the method is suitable for cases where intense monitoring is necessary, for instance fires in the urban-wildland interface where, often, cameras are already installed. The objective of the proposed system is not to measure large wildland fires. The number of cameras and communication infrastructure would be unaffordable for operational conditions. Its extension to large fires is the object of current research. Section 6 contains further discussion on the application of the proposed system for real fires.

By analyzing the previous approaches, the system presented in this paper intends to fill a gap in the current systems in terms of the spatial and temporal resolution of the information obtained. A system of this kind is not intended to substitute other available means but to complement the already existing tools. The use of UAS together with static cameras provides modularity and flexibility, which are suitable properties in highly changing environments [34]. The mobility of UAS can be used to dynamically reduce uncertainty in fire perception, for instance, by taking images from viewpoints complementary to views from fixed cameras. In addition, they enable reacting to changes in the scenario, for instance, by moving to another viewpoint in the case where smoke occludes the images. The authors believe that the main contributions of the work presented in this paper are:

– Exploitation of complementarities between visual and infrared cameras at static locations and mounted on UAS in order to improve the perception in terms of accuracy, resolution, robustness and the capability to adapt to changes in environment conditions.
– Integration of measurements from the cameras available in a statistical framework that adapts the merging process dynamically and is able to continue providing fire estimations if one or more cameras stop working (due to damage by the fire, for example).
– Implementation and validation of the system in close-to-operational conditions in field experiments carried out in Portugal and Spain from 2001 to 2006.

## 3. General Description

**Figure 1.**
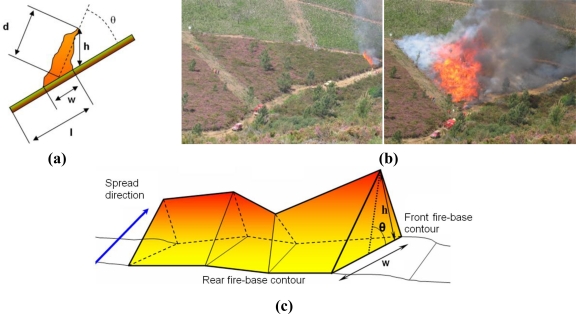
**(a)** Basic scheme of fire geometrical model. **(b)** Visual images taken during a field fire experiment. **(c)** Geometrical model of a fire front.

The main objective of the proposed system is to automatically obtain geometrical measurements of forest fires in real time, such as the location and shape of the fire front, the rate of spread and the fire flame height. A fire geometrical model commonly assumed in the forest-fire domain is depicted in Figure 1(a).

The main fire features in this model can be divided into fire base measurements (fire-front location l, width w) and into flame measurements (length d, height h and inclination angle θ). Two photographs of a fire taken during a field fire experiment in Serra de Gestosa (Portugal) in 2006 are shown in Figure 1(b). For geometry measuring purposes the fire fronts can be approximated by a concatenation of triangles, see Figure 1(c), each of them being characterised by the aforementioned features. The rate of spread is determined by the temporal evolution of the fire-front location.

### 3.1. Sensors

The system uses two main types of imaging sensors with different wavelength bands: infrared cameras and visual cameras, which have interesting synergies for fire perception. Visual cameras provide images in the visible range 0.4–0.7 μm. They can be used to obtain flame measurements (height, inclination angle) applying suitable image processing techniques. They can also obtain fire base measurements (location, width). However, visual images can be occluded by smoke, whose spread is difficult to be predicted in natural outdoor scenarios.

On the other hand, infrared cameras provide images of the scene containing the radiation intensity field within the infrared band. Infrared cameras are not affected by smoke, being highly transparent compared to the high radiation levels originated in a forest fire. The radiation intensity emitted by the base of the fire is considerably higher than that of the flames [35]. Thus, infrared images can be used to obtain measurements of the fire base but are not useful for measuring the flames. Both types of sensors are necessary to obtain the aforementioned fire geometrical model.

The measurement system can use thermal infrared cameras and also non-thermal cameras, which do not provide temperature measurements but do allow qualitative estimations of the radiation intensity. Although cameras in the mid-infrared spectral window 3–5 μm are preferred mainly due to its lower atmospheric absorption, the proposed system can indistinguishably use cameras in the mid-infrared or far-infrared windows. In fact, both types of cameras were used in most of the field experiments that have been carried out.

### 3.2. System Deployment

Perception using distributed cameras increases the robustness of the measurement against potential sources of errors. In some cases, distributed fixed cameras cannot cope with unexpected changes in the spread of fire and smoke. Unmanned Aerial Systems, used as ‘flying cameras’, are ideal platforms on which to overcome these constraints: they can be controlled to move to a suitable viewpoint to improve the observation of the fire or to complement views from fixed cameras. The system presented here exploits the complementarities between the cameras by adopting a statistical sensor fusion approach. Sensor fusion techniques can be used to merge measurements from different sensors in order to obtain overall estimations. Thus, they reduce the influence of errors in measurements and increase the overall accuracy of the system. Statistical data fusion techniques, such as Bayesian Recursive Filters, dynamically adapt the merging process taking into account current errors in the measurements from each of the sensors. The data fusion technique adopted is described in Section 5.

**Figure 2.**
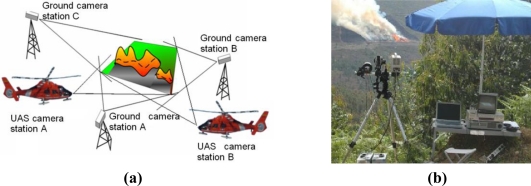
**(a)** A typical deployment of the proposed forest-fire measuring system. **(b)** Picture of a ground station with one *Mitsubishi IRM-300* infrared camera and one visual camera in a fire experiment in Serra de Gestosa (Portugal).

In a typical case, the fire measuring system requires one of more camera stations and one main processing station. Each camera station (fixed on the ground or mounted onboard an Unmanned Aerial System) can be equipped with one camera (visual or infrared) or two cameras (visual and infrared). A deployment with three ground camera stations and two UAS camera stations is depicted in Figure 2(a). A photograph of a ground station with one infrared camera and one visual camera in a fire experiment is shown in Figure 2(b).

If a camera station is deployed with the camera optical axes in the main direction of the fire-front advance (we call them frontal views), visual cameras can be used to obtain measurements of the fire base and of the flames when smoke does not occlude the images. Frontal infrared cameras enable us to obtain fire base measurements. If a camera station is deployed with the camera axes perpendicular to the fire-front spread (lateral views), visual images are useful to determine flame height and inclination angle. In practice deployment constraints can arise due to the topography of the terrain. The viewpoint of cameras onboard UAS can be set to complement static cameras and can be changed dynamically, for instance, if smoke occludes the images. The forest-fire measuring system was designed and developed to be modular and flexible to a wide range of different deployments (fixed/mobile, infrared/visual cameras) suitable for the topography and conditions of each case.

**Figure 3.**
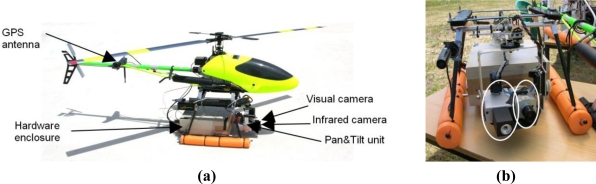
**(a)** Main sensors and components of one helicopter used in forest-fire measurement. **(b)** Detailed picture of the pan and tilt device with one *Raytheon 2000AS* OEM infrared micro-camera and one visual camera.

Aerial images can be used to measure the fire base (location, width and shape) but cannot accurately measure flame height due to the parallax problem. The Unmanned Aerial Systems are equipped with Differential GPS (DGPS) receivers and Inertial Measurement Units (IMUs) so that their location and orientation is known with accuracy. The photograph of a helicopter UAS used in the experiments is shown in Figure 3. It carries a pan and tilt device with one low-cost infrared micro-camera in the far infrared band and one visual camera.

### 3.3. Forest-Fire Measurement Processing

**Figure 4.**
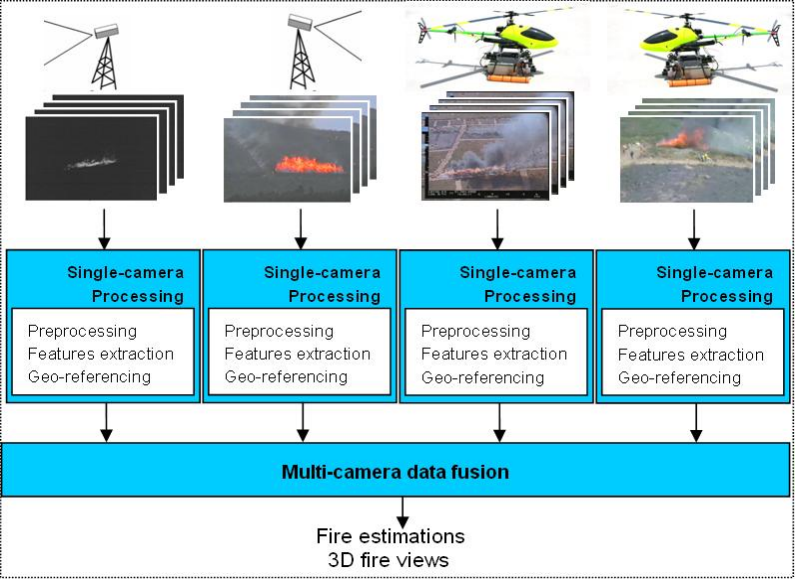
Diagram of the proposed forest-fire measurement system.

A diagram of the main steps in the proposed forest-fire measuring system can be observed in Figure 4. The main inputs of the system are the images from each of the cameras. All the cameras have been internally calibrated using calibration patterns. Each image is associated to data regarding the type of camera, its location and orientation, timestamps and camera calibration information. In the case of cameras onboard an UAS, all the images captured are tagged locally with the composed location and orientation of the aerial vehicle and the pan and tilt unit.

The proposed forest-fire measurement is carried out in two main steps: single-camera processing and multi-camera data fusion. Single-camera processing blocks apply image processing methods to compute fire measurements independently for each of the cameras deployed. This block includes image pre-processing methods to filter out spurious effects and, in the case of UAS, special software dedicated to reject camera vibrations induced by the vehicle. Electro-mechanical systems, such as gimbals, were avoided in our implementation due to the payload constraints of small aerial platforms, such as those employed in the experiments. Instead, it includes stabilization methods based on image processing. Single-camera processing blocks also include image processing methods to extract fire features from the images. Different algorithms are used depending on the type of camera-infrared/visual and fixed/mobile-providing high modularity and flexibility suitable for a wide variety of camera deployments. This block also includes methods to transform these image-plane fire features to real-world measurements. The main techniques used are summarised in the next section.

The multi-camera fusion block integrates the fire measurements computed individually from each camera to obtain overall forest-fire estimations. The data fusion method adopted is based on Kalman Filtering, see Section 5. It implements temporal and spatial filtering techniques to cancel high-frequency fluctuations and local errors. This block also generates 3D views of the fire.

## 4. Single-Camera Processing

This section briefly describes the main techniques used to obtain forest-fire measurements from each camera: image pre-processing, fire feature extraction and image geo-referencing.

### 4.1. Image Pre-Processing

The objective is to increase the quality of the images before extracting fire features. In the first step, a simple 3 × 3 median filter is applied to cancel out image noise and potential spurious effects. Changes in lighting conditions are an important source of error in outdoor computer vision applications. In the forest-fire measuring system the lighting compensation method proposed in [36] is adopted. The main idea is to dynamically modify parameters of the cameras, such as gain and brightness level, in order to keep the lighting conditions in the images gathered constant despite the changes in the environment. The current illumination conditions of the images (essentially brightness and contrast) are measured and compared with the reference-desired-values. The error is used to select suitable lighting parameters of the camera, as in a feedback control scheme.

**Figure 5.**
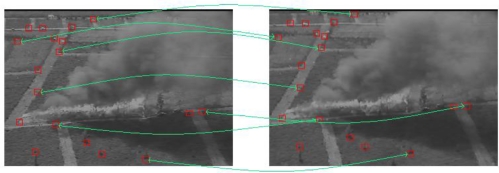
Feature extraction and association in two consecutive aerial visual images.

In addition, the vision-based method described in [37] is used to cancel the vibrations induced by the UAS in the images. Assume *Im_t_*(*x*,*y*) and *Im_t+1_*(*x*,*y*) are two consecutive images in a sequence. The image motion compensation used is applied in three steps: estimation of the motion between *Im_t_*(*x*,*y*) and *Im_t+1_*(*x*,*y*), fitting of the motion to a model and application of the inverted motion model to each pixel in *Im_t+1_*(*x*,*y*). The resulting motion-compensated image, Im_t+1_^*^(*x*,*y*), has no apparent motion with respect to *Im_t_*(*x*,*y*). Motion estimation is based on feature association. Assume that the scene contains enough and sufficiently distributed features perceptible in both images. The corner detector described in [38] is applied to both images. Then, in feature association, each feature from *Im_t_*(*x*,*y*) is the centre of a window that is used as a template for matching over *Im_t+1_*(*x*,*y*). Features are associated based on normalized cross-correlation [39], see Figure 5. Feature association allows us to extract the motion of the features in two consecutive images. However, not all the objects in the images may have the same movement, and features with movements different to the general scene motion originate errors in the motion estimation. These feature associations are considered disturbances and should not be used for motion estimation. A method based on Least Median of Squares is used to identify them [37]. In the next step, valid feature associations are used to fit the motion model. The homography matrix is used to model the motion between images since it can be used to describe the transformations originated by changes in the location and orientation of the camera when the imaged scene can be approximated as a plane [40]. Once the homography matrix has been computed, motion compensation (image warping) is performed by applying the inverse homography matrix to all pixels in *Im_t+1_*(*x*,*y*).

**Figure 6.**
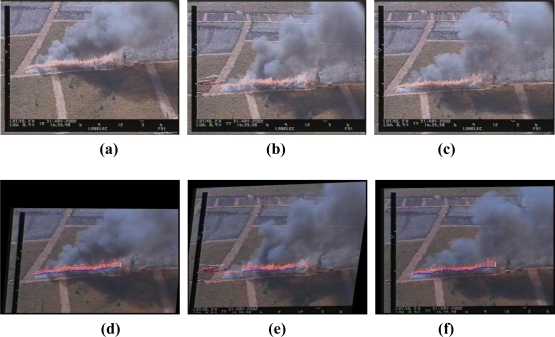
Image stabilization in three consecutive aerial images: **(a–c)** original consecutive aerial images with vibrations, **(d–f)** resulting images after vibration cancellation and extraction of features.

The performance of the image stabilization method is illustrated in Figure 6. Three consecutive aerial images are shown in Figure 6(a–c). Figure 6(d–f) shows the resulting images after vibration cancellation (the image in Figure 6(d) is considered as a reference) and fire feature extraction (described in the next subsection). Notice that the position of the fire front after vibration cancellation is very similar in the three images.

### 4.2. Fire Feature Extraction

Two stages can be identified in this step: fire segmentation and feature extraction. Temperature thresholding is used for segmenting images from thermal infrared cameras. Their temperature measurements depend on parameters, such as the surface emissivity of the object, which in general cannot be known with accuracy in operational fighting conditions. This lack of accuracy has a low influence on image segmentation due to the high difference between fire and background temperatures. On the other hand, non-thermal infrared cameras, such as the *Raytheon 2000AS* onboard the UAS, provide qualitative estimations of the radiation intensity at the scene. In these cases the thresholding algorithm described in [41] is used. As mentioned previously, infrared images are used to obtain measurements of the fire base but cannot be used to measure the flames. Visual cameras are used instead. Fire segmentation in visual images is carried out by a learning-based method similar to that described in [42]. The method requires training images in which a user has determined the pixels that correspond to fire. A histogram is built with the RGB values of each pixel considered as fire in the training images.

**Figure 7.**
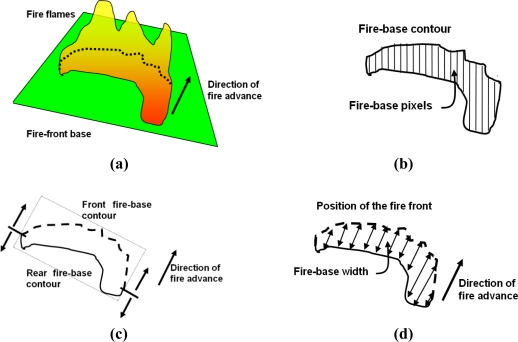
Extraction of features from infrared images: **(a)** fire-front, **(b)** once the fire-base has been segmented, it is easy to identify the fire-base contour, **(c)** taking into account the direction of fire advance, it is simple to differentiate between the front and the rear fire-base contours, **(d)** the fire-base width is computed as the distance between pixels on the front and the rear contours along the direction of fire advance.

From the segmented images it is simple to determine the fire contours. From the fire contours it is possible to determine the fire geometrical features. The extraction of features from infrared images is illustrated in Figure 7. Segmentation of infrared images provides the fire base pixels, whose contour is the fire-base contour, see Figure 7(b). The direction of fire advance can be estimated by analysing the motion of the centroid of the fire-base pixels through time, and then it is possible to distinguish between the front and the rear fire-base contours, see Figure 7(c). Then, fire-base width measurements can be obtained, see Figure 7(d). Similar procedures are applied to visual images. For further details refer to [43].

### 4.3. Image Calibration and Geo-Referencing

The objective is to transform the fire features extracted on the image plane to real world measurements. If the terrain can be locally approximated by a plane, the relation between the terrain plane and the image plane is a homography [44]. A point *P* on the terrain is transformed to point *p* on the image plane of camera *i* using *p* = *H_i_P*, where *H_i_* is the homography matrix for camera *i*, *H_i_* = *A_i_T_i_*, where *A_i_* represents the internal calibration for camera *i* and *T_i_* is the transformation matrix that relates the real-world coordinate system to the coordinate system of camera *i*. The computation of the homography involves establishing correspondences between real-world points on the terrain and their image coordinates. Although the minimum number is four, several well-distributed correspondences are used to reduce the error. In the experiments described in this paper more than 12 were used. Landmarks such as trees and rocks of known size were used to find correspondences. In a real fire application, we do not consider it practical to deploy items only for image calibration purposes. In this case, objects of known location and size that are present in the image, such as fire fighter trucks, could be used. Planar terrain is not a hard constraint. Even though the terrain is not planar, in most cases this assumption is valid if the cameras are located far enough from the fire.

The homography-based method requires planar terrain and a sufficient number of correspondences. If the conditions are not met, the proposed fire measuring system uses the following projection-based method. If a Digital Terrain Model (DTM) of the environment is available, geo-referencing can also be carried out by projecting the image pixels on the DTM. Projection on the terrain requires accurate knowledge of the location and orientation of the camera. In the experiments carried out, they were measured with Differential GPS and Inertial Measurement Units both for cameras on ground stations and onboard UAS. In addition, it requires precise synchronization between images and location and orientation measurements. GPS timing is taken as the reference time for all the sensors. Time stamps are used for all the data to avoid timing confusions.

Further details on both geo-referencing methods can be found in [37].

## 5. Multi-Camera Forest-Fire Estimation

The objective is to integrate all the fire measurements obtained independently from cameras with different perception capabilities. Bayesian Filters provide a well-founded mathematical framework for estimating the state of the system using observations in presence of noise: sensors are modelled as uncertain sources. Decentralised schemes require a strong communication infrastructure (with sufficient bandwidth and coverage) which is often inexistent in wildland environments. In contrast, centralised schemes only require point-to-point communication between each camera station and the main processing station. Adopting a practical approach, in the proposed system we chose the latter option for its easier deployment. In the experiments carried out with up to six distributed cameras, the proposed system was capable of operating on a standard laptop at a rate not lower than 2 Hz, which can be considered real-time for the monitoring of forest fires, whose measurements vary in a clearly larger time scale.

**Figure 8.**
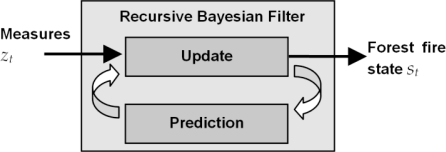
Diagram of the Recursive Bayesian Filter used.

The basic diagram of the Recursive Bayesian Filter (RBF) used is shown in Figure 8 [45]. The input is *z_t_*, the set of measurements obtained individually from each of the *N* cameras. The output of the block is the estimation of the state of the fire front at time *t*, *s_t_*. The RBF requires one update model in order to perform short-term prediction of the evolution of the state and one observation model for each of the cameras used in the deployment. Inaccuracies in the prediction and noise in the observations should be taken into account. RBFs obtain an updated estimation of the state as a weighted average using the prediction of its next state as well as using a new measurement from the sensor. The purpose of this weighting is to give more trust to values with better (*i.e.*, smaller) estimated uncertainty. This process is repeated with every new measurement.

**Figure 9.**
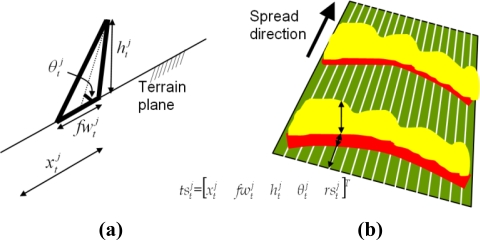
**(a)** Schematic geometry of a fire-front triangle. **(b)** Diagram representing the state of the fire front as the state of a series of equally-spaced fire-front triangles.

As described in Section 3 fire fronts can be approximated by a concatenation of triangles, each of them characterized by the position and width of the fire-base and height and inclination angle of the flames, see Figure 9. The state of the whole fire front is the state of a series of equally-spaced forest-fire triangles, $s_{t}={\left[ts_{t}^{1T}\;\cdots \;ts_{t}^{NT^{T}}\right]}^{T}$, where $ts_{t}^{j}$ is the state of forest-fire triangle *j* at time *t* and *NT* is the number of triangles. The state of a system should contain all the information from the system necessary to predict its future. Thus, $ts_{t}^{j}$ should include the local rate of spread associated to triangle *j*, $rs_{t}^{j},\;ts_{t}^{j}={\left[x_{t}^{j}\;\;fw_{t}^{j}\;\;h_{t}^{j}\;\;\theta_{t}^{j}\;\;rs_{t}^{j}\right]}^{T}$. The state of the entire fire front contains a complete geometrical description of the fire front. Its size is 5 × *NT*. It is possible to select its size by modifying the distance between the fire-front triangles, which can be useful when there are very large fire fronts. The state of two adjacent fire triangles cannot be considered independent. Thus, it is necessary to use one Bayesian Filter for the whole fire front.

Forest-fire modelling is a very complex problem. Forest-fire propagation is subject to the influence of many effects related to terrain topography, meteorology and fuel conditions, among others [46]. A very wide variety of methods and approaches for forest-fire modelling has been researched. The main practical motivation is to use these models in forest-fire attack to predict the behaviour of a fire during the next hours or days, see [47] for instance. Consequently, many of the approaches aim to accurately analyse the phenomena involved in combustion in forest environments, taking into account many factors and thus, resulting in significantly complex models.

It is not the objective of the proposed method to use an exhaustive and highly accurate fire propagation model. The objective in our problem is only to allow the merging of fire measurements in a very short-term prediction-update scheme. In fact, such high accuracy is not needed in our system since the prediction-update cycles of the RBF occur at a frequency not lower than 2 Hz: (1) the prediction error is low for very short-term predictions; (2) the prediction errors are corrected in the update stage of the RBF. The recursive prediction-update scheme of RBFs has been proven to be robust to inaccuracies in the prediction stage [45]: the update stage is capable of contrasting the predictions with the new measurements and of making the suitable corrections.

For measurement merging purposes, our system assumes that, in the very short-term, fire location can be described by a linear dynamic system plus noise in order to take into account the inaccuracies, $x_{t+1}^{j}=x_{t}^{j}+rs_{t}^{j}+w_{1,t}$. The fire base width is related to the rate of spread and the time required for combustion, $fw_{t+1}^{j}=fw_{t}^{j}+a_{1}rs_{t}^{j}+w_{2,t}$, where *α_1_*is used to weigh the contribution of $rs_{t}^{j}$. Flame height depends on the amount of combustible being burnt, which depends on the rate of spread: $h_{t+1}^{j}=h_{t}^{j}+a_{2}rs_{t}^{j}+w_{3,t}$. Also, contiguous fire-front triangles may interact with each other. We describe that interaction in terms of rate of spread, $rs_{t+1}^{j}=a_{3}rs_{t}^{j}+a_{4}rs_{t}^{j-1}+a_{5}rs_{t}^{j+1}+w_{4,t}$, where *α_3_*, *α_4_* and *α_5_* weigh the contribution of the rate of spread at *j*, *j* − 1 and *j* + 1, respectively.

Many effects, such as fuel characteristics and wind conditions, are not considered and would involve important errors in long-term prediction but it is not the case in our problem: we use this prediction only for local short-term updating of the RBF and for measurement merging purposes. Under these conditions, a simple local short-term fire prediction will suffice to integrate fire measurements, as can be observed in the experimental results in Section 6.

The prediction includes temporal and spatial smoothing properties and is capable of cancelling high-frequency fluctuations and local errors in the measurements. From the above expressions, it is easy to obtain a linear representation, *s_t+1_* = *As_t_* + *w_t_*. Many of the entries in *A* are zero, involving moderate computational cost. *w_t_* takes into account these inaccuracies in the prediction, which can be considered to be originated by a high number of independent effects. These errors are assumed to be Gaussian [48]. We will denote the covariance of *w_t_* by matrix *Q*. The parameters *α_1_*, *α_2_*, … *α_5_* and *Q* were set to average values determined by fitting the model with real experimental data.

The observation model for camera *i* follows the expression *z_t,i_* = *C_i_s_t_+v_t,i_*, where *v_t,i_* is the observation uncertainty assumed to be Gaussian with zero mean and covariance matrix *R_i_*. *z_t,i_* is the vector of measurements obtained by camera *i* for all the fire-front triangles, $z_{t,i}={\left[z_{t,i}^{1^{T}}\;\;\;\cdots \;\;\;z_{t,i}^{NT^{T}}\right]}^{T}\;$. The observations of fire-front triangle *j* at time *t* obtained by camera *i* is $z_{t,i}^{j}={\left[x_{t,i}^{j}\;\;\;fw_{t,i}^{j}\;\;\;h_{t,i}^{j}\;\;\;\theta_{t,i}^{j}\right]}^{T}$. *C_i_* can be built using *cc_i_*, (4 × 5 matrix that relates $z_{t,i}^{j}$ and $ts_{t}^{j}$) and *0_4.5_* (4 × 5 zero matrix). *cc_i_* depends on the type of camera. For instance, *cc_1_* in (1) corresponds to an infrared frontal camera that provides fire base location and width measurements while *cc_2_* corresponds to a lateral visual camera that measures flames height and inclination angle:

(1) $$C_{i}=\begin{array}{ccc} \left[\begin{array}{cccc} cc_{i} & 0_{4,5} & \cdots & 0_{4,5}\\ 0_{4,5} & cc_{i} & \cdots & 0_{4,5}\\ \vdots & \vdots & & \vdots \\ 0_{4,5} & 0_{4,5} & \cdots & cc_{i} \end{array}\right] & cc_{1}=\left[\begin{array}{ccccc} 1 & 0 & 0 & 0 & 0\\ 0 & 1 & 0 & 0 & 0\\ 0 & 0 & 0 & 0 & 0\\ 0 & 0 & 0 & 0 & 0 \end{array}\right] & cc_{2}=\left[\begin{array}{ccccc} 0 & 0 & 0 & 0 & 0\\ 0 & 0 & 0 & 0 & 0\\ 0 & 0 & 1 & 0 & 0\\ 0 & 0 & 0 & 1 & 0 \end{array}\right] \end{array}$$

*C_i_* matrices are sparse and involve moderate computational burden. *R_i_* is different for each camera and depends mainly on the camera location and orientation and on the camera type, reflecting the fact that some cameras are more affected by some disturbances, e.g., smoke occlusions, than others. It can also be computed comparing the measurements made with data obtained using photogrammetry.

In our problem, a Kalman Filter is used to implement the RBF. Kalman Filters are suitable for systems with linear prediction and observation and Gaussian uncertainties. The simple prediction and observation models assumed allow efficient implementation. Section 6.2 presents some results that illustrate the advantages of the sensor fusion method proposed.

## 6. Field Experiments

This section is divided into two parts. The first analyses the influence of the main sources of error on fire measurement. The second describes the fire experiments and presents some results.

### 6.1. Sources of Error

The main sources of error in the proposed fire measurement are smoke occluding the visual images, high-frequency fluctuations in the fire front and errors in image geo-referencing. Changes in lighting conditions are compensated for by the method described in [36]. Image synchronization errors can be neglected since fire measurements vary in a clearly larger time scale.

**Figure 10.**
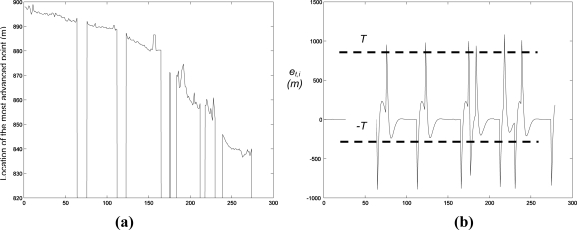
**(a)** Location of the most advanced point of the fire front computed with a visual camera. The presence of smoke prevents the computation of measurements at *t* = 70 s, *t* = 115 s, *t* = 160 s, *t* = 180 s, *t* = 220 s and *t* = 240 s. **(b)** *e_t,i_* throughout the experiment: occlusions are detected.

Smoke occluding the visual images hampers the extraction of valid measurements in visual images. As an example, Figure 10(a) illustrates the effect when computing the location of the most advanced point of a fire-front spreading down-slope. Our system naturally addresses smoke occluding the visual images by using infrared cameras and by deploying cameras at different locations, which helps to reduce the probability of simultaneous occlusion in all the visual cameras.

In addition, UAS can be dynamically controlled to move to locations with good fire perception. Besides, from a data fusion perspective, errors originated by smoke occlusions can be easily detected. Sudden changes in the measurements produce unexpectedly high errors in the update step of the Kalman Filter, *e_t,i_* = *z_t,i_* − *C_i_s_t_*, which can be detected by identifying when |*e_t,i_*| is higher than a threshold *T*, see Figure 10(b). The value of *T* depends on the degree of uncertainty of the measurement. We adopted *T* = *k*√*σ*, where *σ* is the expected variance in case of no occlusion. *k* was experimentally chosen as *k* = 10. A measurement is considered valid if |*e_t,i_*| < *T*. The Kalman Filter only integrates measurements considered to be valid.

The proposed fire measurement system is also robust to the failure of cameras, for instance, in case of the camera being damaged by the fire. Camera malfunctioning originates unexpected changes in the measurements and high errors in the update step of the Kalman Filter. Thus, the aforementioned method also prevents the system from integrating measurements from cameras that are malfunctioning. Of course, in the case of failure, the number of cameras whose measurements are integrated by the fire measuring system decreases.

**Figure 11.**
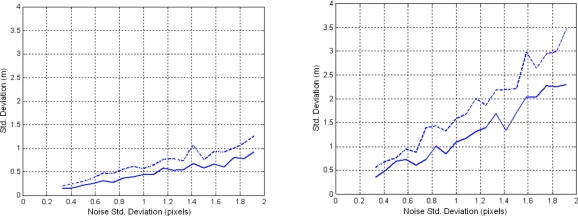
Results of sensitivity analysis in homography-based (left) and terrain projection (right) methods.

To test the influence of the geo-referencing methods several Monte Carlo analyses were conducted by deliberately introducing errors in both geo-referencing methods described in Section 4.3. The error was a Gaussian noise with zero mean and different variances. Fire features extracted in the image plane were geo-located by both error-polluted geo-referencing methods. Some results in two images of the same experiment with frontal (left) and aerial views (right) are shown in Figure 11. In the frontal view, the errors were introduced in the correspondences used for the computation of the homography matrix. In the aerial view, the errors were introduced in the location of the camera. The abscissa axis represents the standard deviation of the noise introduced and the ordinate axis represents the standard deviation of the error in geo-referencing in axes *x* (full line in Figure 11) and *y* (dashed line). The results show that the homography-based method is rather robust to errors in the selection of correspondences, see Figure 11 (left). On the other hand, terrain projection is not as robust to errors in the location of the camera, see Figure 11 (right). To prevent these errors the location of the UAS is measured with Differential GPS (DGPS).

The homography-based method requires planar terrain and a sufficient number of correspondences. If the conditions are not met, the projection-based method is adopted. Although more sensitive to errors, it can be applied if a Digital Terrain Model is available.

### 6.2. Experimental Results

**Figure 12.**
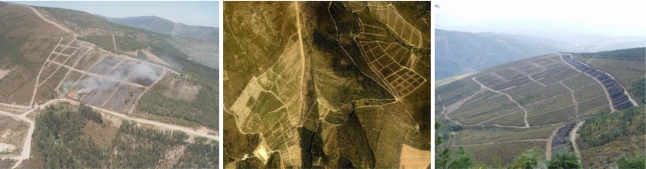
Pictures of the Gestosa experimental site.

The proposed system has been extensively tested in fire experiments carried out in Portugal and Spain from 2001 to 2006. In the experiments, plots of dimensions of up to 160 m × 160 m were burned in controlled conditions [49]. Significant resources were used in these experiments including over 80 firemen, over 5 fire trucks, manned helicopters and several UAS. Three images of the Gestosa site (Portugal) can be observed in Figure 12. The experimental area of Gestosa included more than 60 different plots distributed over several square kilometres. Each year different plots were burnt and different positions of the ground cameras were used. The deployment of the system including the number and type of cameras used and their location was chosen for each experiment taking into account the terrain topography. The forest-fire measuring system was designed and developed to be modular and flexible to a wide range of different camera deployments.

Visual cameras are equipped with motorized zooms. Infrared cameras are equipped with different lenses, which are selected to best fit the image. The visual cameras were *JAI 2060* with a resolution of 752 × 582 and were equipped with zooms with focal lengths from 15 to 90 mm. Four different infrared cameras are used: *Mitsubishi IRM-300* in the mid-infrared spectral window with lenses with a horizontal field of view (hFoV) of 24°, *FLIR 550 Elite* in the mid-infrared window with two lenses (hFoV of 10° and 20°), *FLIR Thermacam P20* in the far-infrared window with three lenses (hFoV of 12°, 24° and 45°) and *Raytheon 2000AS* micro-cameras in the far-infrared window with a 24° hFoV lens was used onboard the UAS.

The proposed system relies on close views of the fire from different viewpoints. Of course, the resolution of the fire measurements obtained depends on the distance from the cameras to the fire and on the lenses used. In real fires, our suggestion is to use zooms for ground stations and to complement them with images from UAS controlled to gather close views of the fires. In the experiments carried out, the system provided reliable results if the fire-front line corresponded to percentages of the image width higher than 18%. As an example, in the experiments performed in year 2002, the ground frontal station was located at approximately 2.9 km. In these experiments the *FLIR 550 Elite* infrared camera with 10° hFoV lenses was used. The spatial resolution of the measurements from the frontal infrared was 0.5 pixels/m. These measurements were merged with others obtained from cameras onboard UAS with close views, resulting in an increase in the resolution of the overall measurements.

In a typical deployment, the images gathered by cameras in static ground stations were transmitted to the main processing station using analogue radio links with directional antennas. For UAS the images were captured and compressed using the lossless JPEG format with onboard electronics and then were transmitted to the main processing station using a long-range radio modem with onmidirectional antennas. In a typical deployment, all the processing modules in the fire measurement system were executed in the main processing station. In some fire experiments, we tested configurations where the single camera processing modules for UAS cameras were executed onboard the UAS, and the results were transmitted to the main processing station for measurement integration. The development of a fully decentralised fire measuring system is the object of current research.

**Figure 13.**
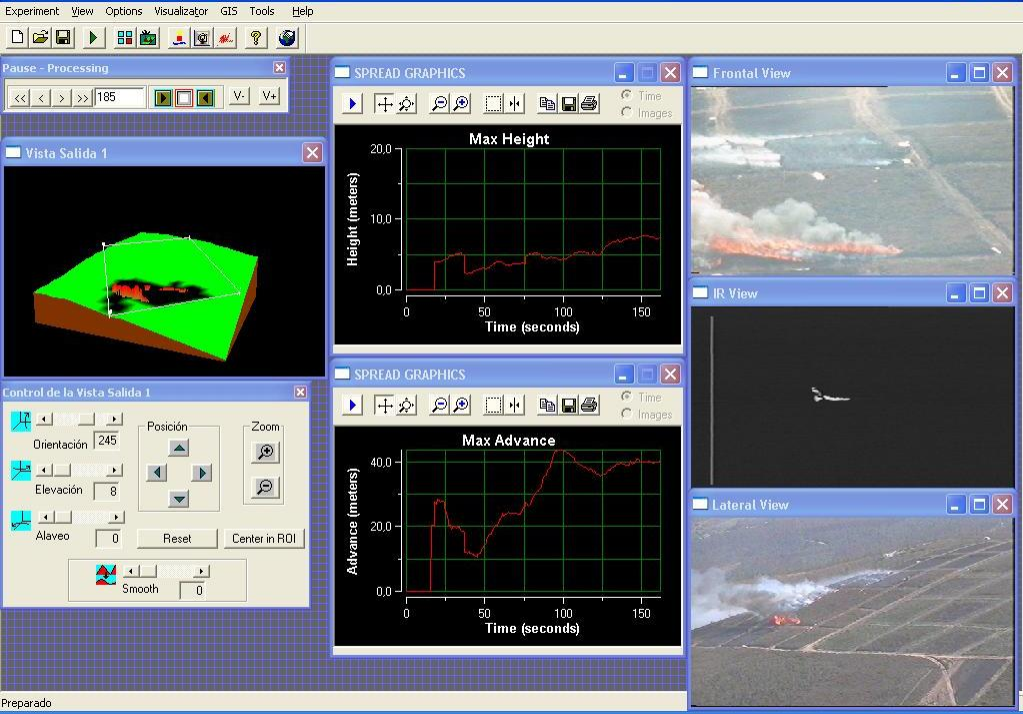
Screenshot of the fire measurement system during an experiment.

**Figure 14.**
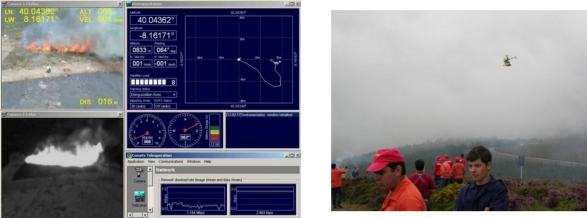
(Left) Screenshot of the UAS station during a field experiment. (Right) Picture taken during the experiments.

Prior to execution, the fire measuring system is configured with data from the deployment, such as the location, orientation and type of the cameras, the number of UAS and the type of the cameras onboard. Figure 13 shows the main window of the fire measuring tool in an experiment with frontal visual and infrared cameras and one lateral visual camera. Images from the three cameras, graphics with some fire measurements and the 3D view of the fire are displayed. A screenshot of the UAS station during an experiment is shown in Figure 14. The images from the visual and infrared cameras onboard and navigation data can be seen. A video of the experiment can be observed in [50]. The videos in [51] and [52] show the processing of aerial visual and fixed visual images in another field experiment. The aerial images are stabilized before segmentation. The videos are generated with a low frame rate to enable the visualisation of results.

**Figure 15.**
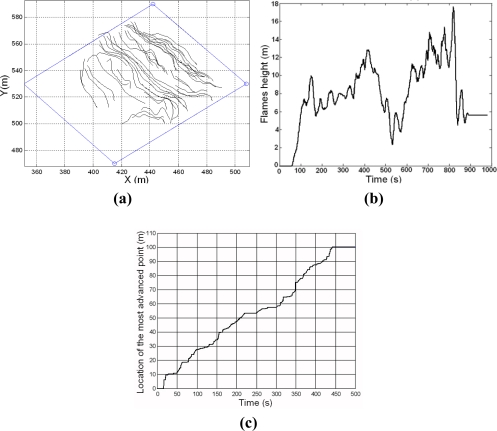
**(a)** Geo-referenced location of the fire front at different times. **(b)** Average flame height of the fire front throughout the experiment. **(c)** Location of the most advanced point of the fire front.

Figure 15 shows some results obtained in a fire experiment: location of the fire front every 30 s in Figure 15(a); the average flame height of the fire front throughout the experiment in Figure 15(b); the location of the most advanced point of the fire front throughout the experiment in Figure 15(c). It should be pointed out that these results were obtained on-site, in real-time, during the fire experiment.

**Figure 16.**
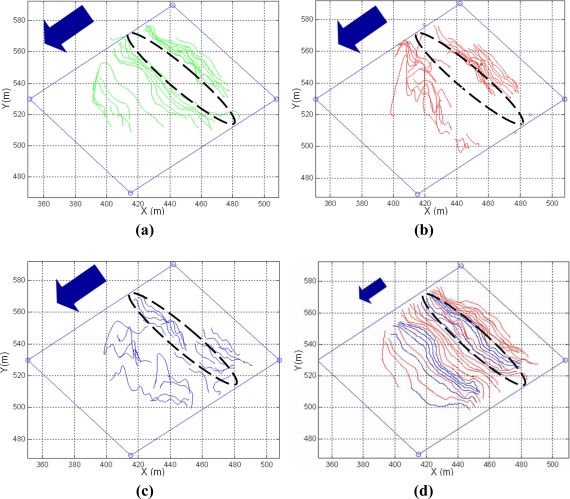
Evolution of the fire-front location in an experiment of a forest fire spreading down-slope, obtained individually from: **(a)** one visual camera onboard an UAS; **(b)** one frontal ground visual camera; **(c)** one ground infrared camera. **(d)** Fire fronts merged from the measurements from the three cameras with the proposed method.

The advantages of using the proposed Kalman Filter method to reduce the errors in fire measurement can be observed in Figure 16. It shows the location of the fire front every 30 s. in an experiment with a forest fire spreading down-slope. Figure 16(a–c) represents the results obtained individually from: one visual camera mounted on an UAS; one frontal ground visual camera; and one frontal ground infrared camera, respectively. The direction of fire advance is represented by an arrow. It can be observed that the fire measurements obtained from the three cameras contain some errors. For instance, during certain periods, the fire produced high levels of smoke and the visual images did not generate measurements, see the dashed ellipses in Figure 16(a–b). The infrared camera is only capable of obtaining valid measurements in this interval.

Figure 16(d) shows the estimations of the fire front after integrating the measurements with the proposed Kalman Filter. The spatial and temporal smoothing capabilities of measurement merging can be noticed. At each time the Kalman Filter integrates all the measurements that are considered valid. Blue fire-front lines in Figure 16(d) represent results estimated by the Kalman Filter with measurements from only one camera. During the smoke occlusion intervals, the Kalman Filter relies on the measurements from the infrared camera. Red fire-front lines represent estimations obtained by merging results from at least two of the three cameras. The overall perception after integrating measurements from different distributed cameras is significantly better than any of the individual perceptions.

**Figure 17.**
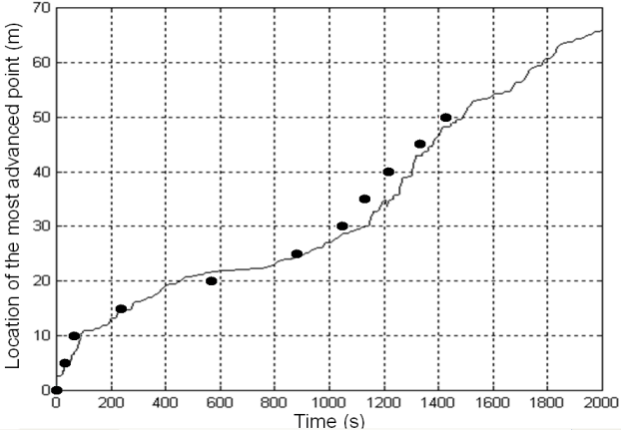
Temporal evolution of the most advanced point of the fire front obtained by the proposed measurement system and its comparison with manual timing.

The proposed system has been compared to classical fire measuring methods consisting in manually timing the intervals in which fires burn through threads placed at regular distances. Figure 17 shows the temporal evolution of the position of the most advanced point of the fire front computed by the fire measurement system. The dots in Figure 17 represent the time at which the fire burns through the threads. The mean relative error between both results is lower than 8%. Similar results were found in other experiments that were carried out.

The experiments showed that the exploitation of the synergies between different cameras located at different positions enables a better estimation of the fire parameters, in terms of accuracy and reliability; overcoming the disadvantages of classical vision-based systems such as occlusion or low resolution. In addition, the data fusion adopted uses the integration of the available information to recover from the short-term failure of cameras. The approach is even able to continue providing fire estimations if one or more cameras stop working (due to damage by the fire, for example). Of course, in this case, the estimated fire parameters will be less accurate due to the lack of this information.

The authors believe that the proposed system is suitable for measuring fires in populated or close to urban areas, where accurate and frequent fire measures are necessary. Fires close to urban areas are usually smaller than those in wildland areas: the lower detection delay and higher availability of extinction means help to prevent the fire from becoming large. In addition, in these areas cameras and communication infrastructure are often already installed and there is good accessibility that allows the quick deployment of the system. The setup makes it difficult to implement the proposed approach in large wildland fires, where the number of cameras and the required communication infrastructures would make the approach unaffordable for operational conditions. However, the main constraints for its use in wildland fires originate in the deployment of the system and not in the image processing or sensor fusion techniques themselves.

Deployment is the main issue for using the proposed system in real forest fires. We do not consider that deploying *ad-hoc* infrastructure to be used in the event of fire is the best option in all cases: the maintenance costs can be high and we might never have a fire. We believe that the best deployment strategy is to benefit from available existing infrastructure and to deploy the required components of the system once the fire has been detected. In case of fire, the system components could be quickly transported in all-terrain trucks. These modified trucks, equipped with cameras on pan and tilt systems, computing and communication resources, could act as mobile ground stations or as processing stations. In case of uncontrolled fires, the trucks and the ground cameras could move to a safer location or with better visibility conditions. On the other, UAS, transported also with the trucks, can be launched manually or using catapults. Although intense implementation work is required to develop such a system, most of the processing and measuring algorithms proposed in this paper remain valid. In addition, it should be noticed that advanced command posts equipped with sensors, computing systems and communications are already being used operationally in forest-fire fighting in many countries, for instance, Spain.

In recent years we have also researched into other solutions based on transportation and self-deployment of loads with several cooperating UAS [53]. The load cannot be transported by only one UAS due to payload constraints and thus, the tight cooperation of several of them is required. These technologies, which have already been tested in urban scenarios [54], could be applied for the deployment of camera stations and communication infrastructure in areas with difficult accessibility. Another option under current research is to extend the approach to a system composed only of cooperating UAS and no ground stations, which would accelerate system deployment and allow us to cover larger fires.

## 7. Conclusions

This paper describes an automatic forest-fire measuring system with cameras fixed at ground stations and mounted on UAS. The method can obtain geometrical forest-fire measurements such as the location and shape of the fire front, fire-base width and flame height, as well as the temporal evolution of these features including the rate of spread, in real-time. The system can provide these fire measurements with significant spatial and frequency resolutions, being suitable for fires of moderate size that require intense monitoring, for instance, those close to urban areas. The objective of the proposed system is not to measure large wildland fires.

The proposed system tackles the sources of error and unpredictable changes in scenario conditions exploiting the complementarities between different types of cameras (visual and/or infrared) fixed at different locations and mounted on UAS. The method is based on obtaining geo-referenced fire measurements individually from each camera and applying statistical data fusion techniques to merge all the measurements into unique estimates. It makes extensive use of image stabilization, fire segmentation, feature extraction, image geo-location and data fusion techniques.

The proposed system has been extensively tested and validated in close-to-operational conditions in field experiments from 2001 to 2006, in which several tenths of hectares were burned under controlled safety conditions. The promising results obtained represent a step forwards to automatic fire measurement in full fire fighting conditions.

This paper opens up several topics of research. UAS have been shown to be very valuable in forest-fire perception. A fire measurement with no ground cameras and only UAS would allow a higher degree of flexibility to adapt to changes in fire and environmental conditions. Fire measurement accuracy increases with the number of cameras deployed. The use of decentralized data fusion techniques in which each agent maintains a perception of the state of the fire would improve the scalability and robustness of the system. Both topics would allow us to cover larger fires and accelerate system deployment. Future work will also explore new mechanisms for system self-deployment in areas with difficult access and without existing infrastructure. In particular, we are analysing the integration of self-deployed ad-hoc sensor networks, ground cameras and aerial robots with sensing capabilities in order to reduce the infrastructure requirements of the proposed approach, enabling it to be applied to extensive wild forest areas.
